# Pep2Path: Automated Mass Spectrometry-Guided Genome Mining of Peptidic Natural Products

**DOI:** 10.1371/journal.pcbi.1003822

**Published:** 2014-09-04

**Authors:** Marnix H. Medema, Yared Paalvast, Don D. Nguyen, Alexey Melnik, Pieter C. Dorrestein, Eriko Takano, Rainer Breitling

**Affiliations:** 1 Department of Microbial Physiology, University of Groningen, Groningen, The Netherlands; 2 Groningen Bioinformatics Centre, University of Groningen, Groningen, The Netherlands; 3 Skaggs School of Pharmacy and Pharmaceutical Sciences and Departments of Pharmacology, Chemistry and Biochemistry, University of California, San Diego, La Jolla, California, United States of America; 4 Manchester Institute of Biotechnology, Faculty of Life Sciences, University of Manchester, Manchester, United Kingdom; University of Canterbury, New Zealand

## Abstract

Nonribosomally and ribosomally synthesized bioactive peptides constitute a source of molecules of great biomedical importance, including antibiotics such as penicillin, immunosuppressants such as cyclosporine, and cytostatics such as bleomycin. Recently, an innovative mass-spectrometry-based strategy, peptidogenomics, has been pioneered to effectively mine microbial strains for novel peptidic metabolites. Even though mass-spectrometric peptide detection can be performed quite fast, true high-throughput natural product discovery approaches have still been limited by the inability to rapidly match the identified tandem mass spectra to the gene clusters responsible for the biosynthesis of the corresponding compounds. With Pep2Path, we introduce a software package to fully automate the peptidogenomics approach through the rapid Bayesian probabilistic matching of mass spectra to their corresponding biosynthetic gene clusters. Detailed benchmarking of the method shows that the approach is powerful enough to correctly identify gene clusters even in data sets that consist of hundreds of genomes, which also makes it possible to match compounds from unsequenced organisms to closely related biosynthetic gene clusters in other genomes. Applying Pep2Path to a data set of compounds without known biosynthesis routes, we were able to identify candidate gene clusters for the biosynthesis of five important compounds. Notably, one of these clusters was detected in a genome from a different subphylum of *Proteobacteria* than that in which the molecule had first been identified. All in all, our approach paves the way towards high-throughput discovery of novel peptidic natural products. Pep2Path is freely available from http://pep2path.sourceforge.net/, implemented in Python, licensed under the GNU General Public License v3 and supported on MS Windows, Linux and Mac OS X.

This is a *PLOS Computational Biology* Software Article.

## Introduction

After a steady decline at the end of the last millennium, natural products are back in the centre of attention as leads for drug discovery [1]–[4]. The secondary metabolites from which they are derived can be categorized into various classes, according to their chemical structures and the different ways in which they are synthesized enzymatically. Two of the most abundant classes are the NonRibosomally synthesized Peptides (NRPs) and Ribosomally-synthesized and Post-translationally-modified Peptides (RiPPs). Many NRPs and RiPPs are of great clinical and societal importance, having applications as antibiotics, immunosuppressants or cytostatics [5].

Unlike RiPPs, NRPs are synthesized not by the ribosome but by large enzymatic complexes called nonribosomal peptide synthetases (NRPSs). These NRPSs form assembly lines of modules, which each add one specific amino acid to the growing peptide chain [6]. Each NRPS module normally consists of at least three domains: a condensation (C) domain that catalyses the condensation to the next amino acid, the adenylation (A) domain that selects the amino acid to be incorporated, and the thiolation (T) domain to which the growing peptide chain is attached. Because they function independently of the ribosome, NRPSs can introduce not only proteinogenic but also nonproteinogenic amino acids into the peptides they produce. After its synthesis by NRPSs, the core peptide scaffold of an NRP can be further modified by a wide variety of tailoring enzymes.

For a long time, drug discovery from bioactive peptides depended on laborious identification and characterization of one peptide at a time. Moreover, re-discovery of already known peptides occurred with increasing frequency, which made the entire process slow, inefficient and costly [7]. Conversely, the genomic identification of biosynthetic gene clusters (BGCs) that could potentially encode the enzymatic machinery to make novel bioactive peptides is rapidly becoming easier, as thousands of genomes are being sequenced each year and various algorithms have been developed to automatically detect the BGCs that encode NRP and RiPP biosynthesis [8]–[13].

Recently, a new technology, peptidogenomics, has been introduced, which uses the potential of high-throughput mass spectrometry (MS) [14] to dramatically speed up the discovery of novel bioactive peptides [15]. Using this technology, short amino acid sequence tags (which represent a part of the complete peptide) can be reconstructed from the MS peak patterns by looking at the mass differences between peaks of various peptidic fragments. In turn, these ‘mass shift sequences’ can be assessed for their potential to represent novel peptides using dereplication tools such as iSNAP [16] and matched to BGCs predicted by methods such as antiSMASH [10], [11]. So far, the matching of mass shift sequences to BGCs has remained a tedious and complicated procedure, especially for NRPs. In this procedure, possible amino acid sequences are manually compared to substrate specificity predictions of NRPSs by algorithms such as NRPSPredictor2 [17], after identification of NRPS gene clusters by antiSMASH. This lack of automation has severely impeded high-throughput peptidogenomic experimentation, and has precluded the use of peptidogenomics on microbial communities with large metagenome datasets. Moreover, the effective use of peptidogenomics on unsequenced strains [18] also depends on the development of computational approaches, in order to be able to compare identified sequence tags with dozens or even hundreds of genomes of related genome-sequenced strains to identify orthologous BGCs. Here we fill this gap by introducing Pep2Path, a set of algorithms that facilitate the rapid and automatic identification of candidate BGCs that correspond to NRP- and RiPP-derived mass shift sequence tags detected by mass spectrometry. Moreover, we show how Pep2Path can be used to detect BGCs for previously characterized NRPs for which the biosynthetic enzymes had not been identified yet.

## Design and Implementation

### Matching nonribosomal peptide BGCs to tandem mass spectra

**Figure 1:**
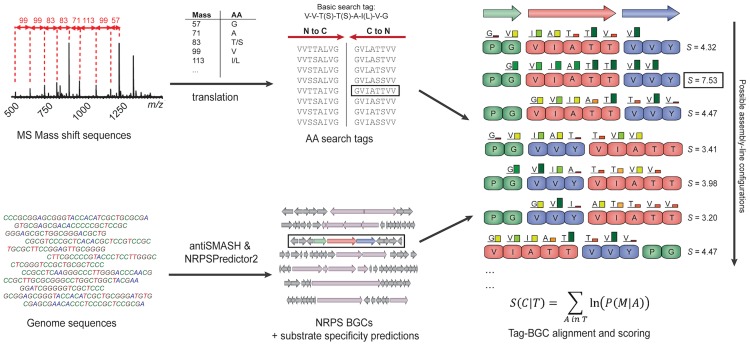
Outline of the NRP2Path matching process. The input for NRP2Path consists of mass shift sequences (or amino acid search tags) on the one hand, and genome sequences on the other hand. The latter are processed into databases by *makedb*, using antiSMASH and NRPSPredictor2. When a database is queried with a mass shift sequence or amino acid search tag, Pep2Path scores all possible matches between search tags and all possible assembly line configurations of each of the NRPS BGCs in the database.

The Pep2Path suite consists of two algorithms, NRP2Path for NRPs and RiPP2Path for RiPPs. The NRP2Path algorithm has to solve by far the more challenging problem. In order to assess how likely it is that a given MS-derived NRP mass shift sequence originates from a certain BGC, NRP2Path uses a Bayesian algorithm to estimate the probability for each amino acid in the tag to originate from each of the NRPS modules of a BGC that encodes a certain NRPS assembly-line (**Figure 1**, see paragraph below for details). Of course, sequence tags can be aligned to a BGC-encoded NRPS assembly line in multiple ways, and BGCs that encode multiple NRPSs may have various possible assembly-line configurations. Hence, the final score for a match between a BGC and a sequence tag will be the maximum score obtained for any of the possible alignments of a sequence tag with each of the possible assembly line orders of the NRPSs encoded by a BGC. To help interpret cases in which multiple NRPS BGCs receive similarly high scores, NRP2Path also calculates a colinearity index, which measures the proportion of contiguous NRPS module pairs that are colinear with the order of amino acids in the identified sequence tag.

### NRP2Path algorithm

Using Bayes' Theorem, the probability *P*(*M*|*A*) that an NRPS module *M* is responsible for the biosynthesis an observed amino acid *A* in a peptide sequence tag *T* is:
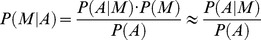
For most applications, the prior probability *P*(*M*) of a module *M* to synthesize any observed amino acid will be the same for all modules and can be neglected. We do note that there are scenarios where the value of this prior probability would become relevant. For example, if one studies peptidic compounds from a microbial community, the abundance of the various species in the community would largely determine *P*(*M*). In such a case, one might estimate *P*(*M*) using the metagenomic coverage of each of the observed species from which genome information is available.

The score *S* for an entire gene cluster *C* given a sequence tag *T* is then the sum of the log likelihoods *P*(*M*|*A*):
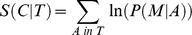
To calculate *P*(*M*|*A*), the probability of an amino acid to be part of an observed peptide *P*(*A*) is estimated using the frequencies of amino acids in known NRPs from the NORINE database. At those positions in a search tag that represent multiple possible amino acids, a baseline value of (1/AA alphabet size) is used for *P*(*A*). This is done in order not to introduce an inappropriate bias towards matches to rare amino acids, which would otherwise lead to high scores because the possible prior observation of such an amino acid would erroneously be treated as an actual prior observation. We use a pseudocount *k* of 1 to correct for the limited sample size. The formula to calculate *P*(*A*), in which 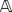 represents the NRP ‘alphabet’ considered, is:
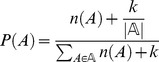

*P*(*A*|*M*) is estimated as a function of the baseline probability *P*(*A*) and a variable *I_A,M_*, which is determined by the two types of NRPSPredictor2 substrate specificity predictions for the A domain in the module. Hence, the *I_A,M_* variable is an average value of two *I* values: one for the support vector machine matches and one for the Stachelhaus code matches for an NRPS module *M* to an amino acid *A*. For the Stachelhaus code predictions, the value of *I* corresponds to the degree of identity of the Stachelhaus code of an NRPS module (10 amino acids long) to that of the most closely related known NRPS module within the NRPSPredictor2 search space. For the SVM predictions, *I* will be 1.0 if the amino acid in the sequence tag matches the single amino acid prediction for the NRPS module, 0.75 if the amino acid matches the small class prediction, 0.5 if the amino acid matches the large class prediction, 0.25 if the amino acid matches the three-class prediction, and 0.0 if the amino acid matches none of these. *P*(*A*|*M*) is calculated with the following formula, in which *c* is a confidence factor (default value = 1) that accounts for how much of the final probability is determined by the substrate specificity predictions, as compared to the contribution by the baseline probability *P*(*A*), and *x* is again a pseudo-count variable to correct for finite sample size, this time considerably smaller than 1 (the default settings of the Pep2Path software use *x* = 0.01):
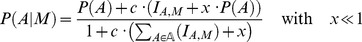



Finally, we can also add an η variable that allows NRPSPredictor2 mismatches to be penalized exponentially as the number of mismatches increase:
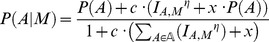
The default value in the Pep2Path software is *η* = 2.

Combining the equations, we obtain
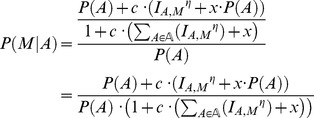
from which the score *S*(*C*|*T*) can be calculated directly:
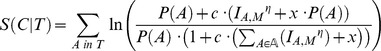
Of course, sequence tags can be aligned to a BGC-encoded NRPS assembly line in multiple ways, and BGCs that encode multiple NRPSs may have various assembly-line configurations. Hence, the final score for a match between a BGC and a sequence tag will be the maximum *S* score obtained for any of the possible alignments of a sequence tag with each of the possible assembly lines encoded by a BGC.

### Constructing and merging NRP2Path databases

The input of genomic data to which NRP sequence tags can be matched proceeds through the construction of NRP2Path databases. For this purpose, two accessory programs, *makedb* and *mergedb*, are provided with Pep2Path. The *makedb* tool uses antiSMASH2 [11] to search user-provided input sequences for BGCs that encode NRPSs and integrates information on these BGCs into a database. Each entry in this database consists of the accession number or name of the nucleotide entry from which the BGC originates, a list of genes that constitute the BGC, taxonomic information on the species whose genome encodes it, the modular architecture of the NRPSs within the BGC, and substrate specificity predictions as given by the two NRPSPredictor2 algorithms [17]. A database with all NRPS-containing BGCs within the GenBank database is already provided with Pep2Path. The *mergedb* tool can be used to merge this database with custom-made databases created from locally available sequence data, or to combine multiple custom-made databases with one another.

### Input data for NRP2Path

The input for NRP2Path is either a list of amino acids or a list of mass shifts. In the latter case, Pep2Path converts the mass shifts into amino acid sequence tags using the conversion table provided by Kersten et al. [15]. Because some amino acids or amino acid derivatives have identical masses, NRP2path will generate a list of all possible short peptide sequences. Then, NRP2path assesses for each of the BGCs within the selected NRP database how likely it is that this BGC encodes the peptide from which the sequence tag derives (**Figure 1**). Depending on how much is known about the source of the biological material analyzed by MS, the user can select a taxonomic range (strain, species, genus, etc.) within which to search.

### RiPP2Path: a simple accompanying tool to match RiPPs to prepeptide-encoding genes

Besides matching of NRPs to their BGCs, the Pep2Path suite also offers the ability to match RiPPs to the prepeptide-encoding genes that encode their primary sequences, similar to the matching module from the recently launched RiPPquest [19]. This is done by the RiPP2Path algorithm. Like NRP2Path, RiPP2Path uses a dedicated translation table to convert an identified mass shift sequence into all possible amino acid sequences that it could represent. To match these possible RiPP amino acid sequences to genomically encoded precursor peptides, it then reads in a set of (meta)genomic sequences and retrieves their six translation frames. Finally, it performs a simple matching of each possible RiPP sequence to the translation frames, using a sliding window with a word size equal to the tag length. The key difference between the RiPPquest and RiPP2Path algorithms is that RiPPquest (in its current version) is specifically aimed at lanthipeptides, while RiPP2Path can identify prepeptides of any type: hence, RiPPquest will be better suited to search for lanthipeptides in a large number of genomes, while RiPP2Path will be the method of choice for discovering other (and novel) types of RiPPs. Also, unlike RiPPquest, which uses molecular networking to locate matches to a database, RiPP2Path directly uses mass shift or amino acid sequences as input.

## Results

### Benchmarking shows that NRP2Path is powerful and robust

**Figure 2:**
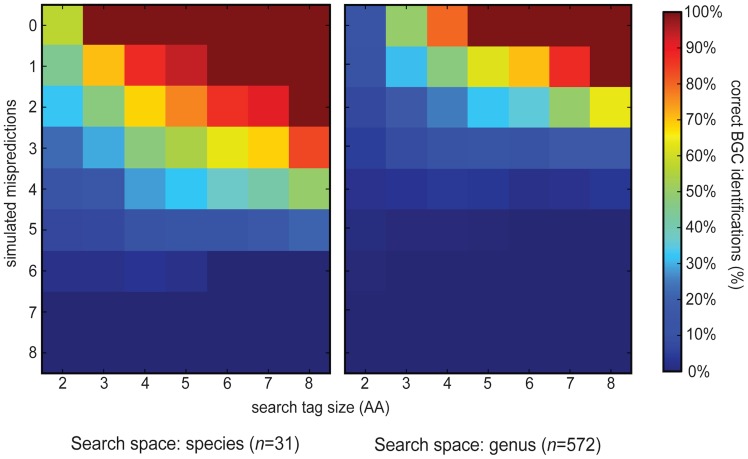
Quality of NRP2Path predictions with varying sequence tag lengths and NRPSPredictor2 prediction qualities. The heat map shows the average number of correct BGC predictions for Pep2Path searches with the stendomycin sequence tag V-V-T(S)-T(S)-A-I(L)-V-G across the *Streptomyces hygroscopicus* ATCC 53653 genome (20 NRPS BGCs) or across all *Streptomyces* nucleotide entries (342 NRPS BGCs). The searches were done for all possible search subtags of 2–8 amino acids long, and for all combinations of 0–8 simulated mispredictions for the corresponding NRPS modules. Mispredictions are simulated with zero scores given by Pep2Path for sequence tags matching to these domains.

As a first assessment of how NRP2Path would confront the difficult challenge to match NRP-derived mass shift sequences to BGCs, we tested whether it would be successful in matching the V-V-T(S)-T(S)-A-I(L)-V-G sequence tag that was used to identify the BGC of the nonribosomal peptide stendomycin by Kersten et al. [15]. Pep2Path appeared to do this very effectively: even when the search space consisted of all 12,470 NRPS BGCs in the GenBank database, the stendomycin BGC emerged as the top hit. By varying the sequence tag size between 2 and 8 and introducing 0–8 simulated mispredicted substrate specificities (by artificially setting the score for a query amino acid to zero) for the corresponding stendomycin NRPS modules, we show that also BGCs with less accurate NRPSPredictor2 predictions (i.e., with up to one or two completely wrong predictions) can be identified robustly within a genome or species, even with relatively small sequence tags (**Figure 2**).

### NRP2Path benchmarking on 18 NRPs of known structure

To investigate how well NRP2Path would work in practice on novel compounds, we mined 18 NRPs from the recent scientific literature, together with their corresponding NRPS BGCs (**Table S1**). None of these had yet been incorporated into the NRPSPredictor2 training sets. In order to assess how well Pep2Path would be able to match tags from these NRPs to the correct BGCs under varying conditions, we varied the sequence tag size from 2 to 8 and tested all possible search tags of these sizes on databases with sizes ranging from 5 NRPS BGCs to 100 NRPS BGCs (an average bacterial genome contains ∼5 NRPS BGCs). For each database size, 50 randomly permuted BGC databases were created from BGCs originating from genomes within the same (sub)phylum, and the results were averaged across all of these permutations. The results (**Table 1**) confirm that the minimum sequence tag size to confidently match an NRP to a BGC is around 2–4 when the genome sequence is known. When the search space is larger, a situation that represents the mining of unsequenced strains for NRPs and attempting to match them to orthologous BGCs within the same genus [18], larger sequence tags (e.g., 5–8 amino acids long) are often still sufficient to identify the correct BGC.

**Table 1:** Benchmark of Pep2Path on 18 recently discovered NRPS BGCs.

Tag size (AA)	BGC search space size: 5	BGC search space size: 10	BGC search space size: 25	BGC search space size: 50	BGC search space size: 100
2 (*n* = 18)	75%	64%	47%	36%	26%
3 (*n* = 15)	78%	70%	54%	44%	37%
4 (*n* = 12)	83%	78%	65%	56%	45%
5 (*n* = 11)	90%	89%	79%	72%	61%
6 (*n* = 8)	96%	96%	87%	81%	74%
7 (*n* = 5)	99%	99%	96%	91%	88%
8 (*n* = 3)	100%	100%	100%	100%	100%

For each tag size, all possible search tags of that size in the test set of peptides (**Table S1**) were used as queries. For each BGC search space size, 50 search spaces were generated from randomly selected BGCs from the same (sub)phylum that the NRP originates from. The resulting percentages represent the average number of cases in which the correct BGC ended up as the (shared) best hit across all possible sequence tags and across all possible search space permutations. Shared best hits were included because of the frequent presence of orthologous BGCs encoding the same molecule in related genomes. The *n* in the left column signifies the number of test peptides large enough to be included in the analysis for this tag size; from each of these test peptides, all possible subtags were used in cases where the length of the tag is shorter than the length of the peptide.

### New gene clusters for old molecules: an application

Finally, we used NRP2Path in an effort to find BGCs for NRPs within the NORINE database [20] for which no BGC had been discovered so far. Intriguingly, we discovered novel candidate BGCs for four molecules by searching all NRPS BGCs from the species from which the compound had originally been isolated (**Table 2**). When we expanded the search space to screen the entire database, we discovered another very good match, for tripropeptin A (**Table 2**). Surprisingly, although this eight-amino-acids-long peptide was originally discovered in the gamma-proteobacterium *Lysobacter* sp. [21], we found a match with a BGC in the genome of the beta-proteobacterium *Collimonas fungivorans* Ter33 (**Figure S1**). The tripropeptins are highly important molecules from a pharmaceutical point of view, as tripropeptin B and C display potent antibacterial activity against methicillin-resistant *Staphylococcus aureus* (MRSA), vancomycin-resistant enterococci (VRE), and penicillin-resistant *Streptococcus pneumoniae* [22], [23]. All in all, the results of the NORINE searches show how even in the absence of new peptidogenomic data Pep2Path can identify putative genomic loci that encode the biosynthesis of important molecules, opening up new possibilities to further engineer these pathways and/or produce the compounds in high titers through metabolic engineering or heterologous expression.

**Table 2:** Novel matches of NORINE-derived NRPs to BGCs detected in genome sequences.

Compound	Reference	Species (accession nr.)	Locus tags	NRP search tag from NORINE	NRPSPredictor2 prediction	Pep2Path score (rank)
trichotoxin	(Irmscher et al. 1978)	*Trichoderma virens* Gv29-8 (ABDF02000085)	TRIVIDRAFT_69940	ala-gly-ala-leu-ala-glu-ala-ala-ala-ala-ala-ala-pro-leu-ala-xxx-gln-vol	nrp-nrp-ala-nrp-nrp-gln-nrp-ala-nrp-ser-leu-nrp-pro-nrp-ala-ala-gln-vol	6.25 (1)
ferintoic acid	(Williams et al. 1996)	*Microcystis aeruginosa* 9701 (CAIQ01000336)	MICAK_4000004-MICAK_4000007	trp-co-lys-val-hty-ala-phe	phe-nrp-lys-val-nrp-ala	5.24 (1)
plusbacin	(Shoji et al. 1992)	*Pseudomonas putida* ND6 (CP003588)	YSA_0461-YSA_0481	asp-pro-ser-asp-arg-pro-ala-allothr	asp-ser-ser-asp-nrp-nrp-nrp-thr	4.91 (1)
amphibactin B	(Martinez et al. 2003)	*Vibrio tubiashii* NCIMB 1337 (AHHF01000067)	VT1337_12727-VT1337_12732	orn-orn-ser-orn	orn-orn-ser-orn	2.73 (1)
tripropeptin A	(Hashizume et al. 2001)	*Collimonas fungivorans* Ter33 (NC_015856)Originally found in *Lysobacter* sp.	CFU_2182-CFU_2185	thr-pro-pro-arg-asp-ser-pro-asp	thr-pro-pro-orn-asp-ser-pro-asp	8.94 (1)

Candidate BGCs for trichotoxin, ferintoic acid, plusbacin and amphibactin B were discovered by searching within the taxonomic range of the species in which the molecules were found. The candidate BGC for tripropeptin A was discovered by searching the entire Pep2Path database.

### RiPP2Path is able to specifically detect RiPP prepeptides in the majority of cases

When testing RiPP2Path on the nine *Streptomyces* RiPPs identified by Kersten et al. [15], seven of these produced unique matches of their search tags to the corresponding genome sequences (**Table 3**). The remaining two search tags could still be manually matched to gene clusters based on the antiSMASH-based identification of RiPP BGCs containing RiPP2Path matches.

**Table 3:** Matching of mass sequence tags to RiPP gene clusters using RiPP2Path.

Peptide	Search tag	Genome	Matches in genome
SSV-2083	I(L)GA(C)GTA(C)WI(L)A(C)V	*Streptomyces sviceus* ATCC 20983	1
SGR-1832	AVAQ(K)FVI(L)Q(K)GSTI(L)	*Streptomyces griseus* IFO 13350	1
SCO-2138	VHFVGWI(L)	*Streptomyces coelicolor* A3(2)	1
SLI-2138	GI(L)VHFVGWI(L)	*Streptomyces lividans* TK24	1
SWA-2138	I(L)AGI(L)VHFI(L)GWI(L)	*Streptomyces* sp. E14 (WASP)	1
SRO15-2005	YWSRRI(L)I(L)	*Streptomyces roseosporus* NRRL 15998	1
SRO15-2212	VVI(L)S(C)T	*Streptomyces roseosporus* NRRL 15998	47
SRO15-3108	AS(C)ATVTI(L)	*Streptomyces roseosporus* NRRL 15998	1
SAL-2242	VTI(L)S(C)T	*Streptomyces albus* J1074	39

Seven out of the nine search tags resulted in unique matches in their corresponding *Streptomyces* genomes.

## Availability and Future Directions

Pep2Path is freely available from http://pep2path.sourceforge.net/, implemented in Python, licensed under the GNU General Public License v3 and supported on MS Windows, Linux and Mac OS X. To maximize the convenience for the majority of experimental scientists in using a new computational method, it is often helpful to provide a user-friendly web-server with accompanying step-by-step protocols for standard analytical scenarios. Providing these for the Pep2Path tools is one of the remaining challenges for future development of the software.

All in all, the automated peptidogenomics technology offered by Pep2Path constitutes a radical departure from the one-molecule-per-study approach to drug discovery from natural products that has dominated the field for long. The combination of the rapid sampling of chemical space by tandem mass spectrometry with effective computational matching of the chemistry to BGCs will allow quick identification and dereplication of large numbers of novel peptides. While detailed structural characterization of the hits for now remains a bottleneck, new methods are appearing that will accelerate this process as well [24]. For now, identified peptides can be prioritized for further detailed characterization based on the biosynthetic novelty inferred from the BGC architecture, combined with phenotypic assays.

With nanoDESI mass-spectrometry [25], automated peptidogenomics also allows genome mining directly from environmental samples, without strain isolation or genome sequencing: the identified peptides can be matched directly to gene clusters in the rapidly growing database of already sequenced genomes [18]. When combined with large-scale metagenomics, automated peptidogenomics will also make it possible to sample NRPs in environmental samples at large scales, by integrating mass spectral molecular networks [26], [27] from MS data with BGC similarity networks [28], and cross-linking these two network types with a third type of networks that represent Pep2Path matching scores between molecule families and gene cluster families.

The key limitation of Pep2Path is that the quality of its results depends on the accuracy of the substrate specificity predictions by NRPSPredictor2. For some organisms, particularly fungi, the training data for these predictions is currently still limited, which leads to a decrease in predictive power. Hence, we will strive to continuously update the dataset of experimentally verified NRPS adenylation domain substrate specificities according to rigorous data standards, in order to keep improving the power of approaches such as Pep2Path as knowledge progresses.

The approach described here can potentially be extended to other compound classes, such as saccharides [29], once an automated system for the detection of sugar biosynthesis genes and prediction of glycosyltransferase substrate specificities has been set up. Hence, Pep2Path paves the way for the high-throughput characterization of the vast universe of BGCs discovered in genomic data of organisms throughout the tree of life.

## Supporting Information

Code S1 **Source code and test data.** Full source code of Pep2Path version 1.1.0 and associated test data that allow users to test the functionality of the software. Updated versions of the Pep2Path code will be made available on http://pep2path.sourceforge.net in the future.
(GZ) Click here for additional data file.

Figure S1 **Gene cluster and predicted possible biosynthetic scheme for the production of tripropeptin-like molecules by** ***Collimonas fungivorans*** **Ter331.** The gene cluster was identified by Pep2Path based on the raw amino acid sequence of compounds originally purified from *Lysobacter* sp. BMK333-48F3. The outline of the gene cluster and its encoded NRPS assembly-line above shows that the architecture of the gene cluster is consistent with the chemical structure of molecules highly similar to the tripropeptins that have been identified in *Lysobacter*. This BGC has eight NRPS modules, of which seven gave NRPSPredictor2 predictions exactly matching the tripropeptin A sequence in the right order, while the eighth prediction was only a near miss (ornithine predicted instead of arginine). This indicates that such a gene cluster might have undergone horizontal gene transfer at least once, from one subphylum to another. The peptides actually produced by *Collimonas* might have small differences in chemistry, compared to the *Lysobacter* tripropeptins, due to slight variations and/or promiscuity in the tailoring reactions and substrate acceptance.
(PDF)

Table S1 **NRP2Path benchmarking dataset.** The table displays 18 recently experimentally characterized NRPs and their biosynthetic gene clusters used for benchmarking NRP2Path. Rare amino acids that are not covered by the Pep2Path translation table are marked as ‘Xxx’.
(PDF)

Text S1 **Pep2Path documentation.** Full documentation for the Pep2Path software, including installation instructions, information on general use, instructions on command-line usage and the software license.
(PDF)

Text S2 **Pep2Path test data documentation.** Description of the comprehensive set of test data that showcases the Pep2Path software. This includes instructions for testing NRP2Path using the stendomycin sequence tag and the set of 18 recently discovered NRPs mentioned in the manuscript text, as well as instructions for testing RiPP2Path using the set of nine recently discovered RiPPs.
(PDF)
