# Supplementary material for: Pep2Path: Automated Mass Spectrometry-Guided Genome Mining of Peptidic Natural Products
Source: PLoS Comput Biol. 2014 Sep 4;10(9):e1003822. doi: 10.1371/journal.pcbi.1003822 (PMC4154637; doi:10.1371/journal.pcbi.1003822)
Supplement: Code S1 — Source code and test data. Full source code of Pep2Path version 1.1.0 and associated test data that allow users to test the functionality of the software. Updated versions of the Pep2Path code will be made available on http://pep2path.sourceforge.net in the future. (GZ) [file pcbi.1003822.s001.gz › TestData/TestData_Instructions.pdf]

PEP2PATH TEST DATA SETS

1. The stendomycin sequence tag

As a first way to test the capabilities of Pep2Path, you can test the stendomycin extended amino acid tag V-V-T(S)-T(S)-A-I(L)-V-G to search the GenBank-wide Pep2Path database at different taxonomic levels, as is shown in the manuscript.

To search for such a extended tag, you can call nrp2path.py as follows:

```
python nrp2path.py --db genbank.ppd
Val,Val,Thr/Ser,Thr/Ser,Ala,Ile/Leu,Val,Gly
```

When searching throughout the entire set of NRPS gene clusters without taxonomic specification, this will yield the following results (top 5 shown), with the biosynthetic gene cluster (BGC) for stendomycin (GG657754_c10) as the best match with a Nrp2Path score of 7.54, compared with scores between 7.47 and 5.79 for hits ranked 2nd to 5th:

```
Sequence tag 1: Val,Val,Thr/Ser,Thr/Ser,Ala,Ile/Leu,Val,Gly
Rank     Accession        Cluster nr       Description      Nrp2Path Score   Colinearity Score
         Sequence tag     Matching AA      Matching domains NRPSPredictor2 code predictions
         NRPSPredictor2 SVM predictions
1.       GG657754 c10     Streptomyces hygroscopicus ATCC 53653 genomic scaffold supercont1.1,
whole genome shotgun sequence.    7.53553517418    1.0
         Val,Val,Thr/Ser,Thr/Ser,Ala,Ile/Leu,Val,Gly        gly,val,ile/leu,ala,thr,thr,val,val
         EFL21631_A1,EFL21631_A2,EFL21631_A3,EFL21631_A4,EFL21631_A5,EFL21631_A6,EFL21631_A7,EFL216
32_A1    gly,val,N/A,ala,thr,thr,val,val   gly,val,val,ala,thr,thr,val,val
2.       AMXK01000076     c1       Pseudomonas syringae BRIP34876 scaffold37_contig1, whole genome
shotgun sequence.7.47385233925    0.8      Val,Val,Thr/Ser,Thr/Ser,Ala,Ile/Leu,Val,Gly
         gly,val,leu,ala,ser,ser
         ELP96332_A5,ELP96332_A6,ELP96332_A7,ELP96332_A8,ELP96326_A1,ELP96326_A2
         ala,val,leu,ala,ser,ser   ala,val,leu,ala,ser,ser
3.       CM001763 c6      Pseudomonas syringae pv. syringae B64 chromosome, whole genome shotgun
sequence.        6.35111708574    1.0      Val,Val,Thr/Ser,Thr/Ser,Ala,Ile/Leu,Val,Gly
         gly,val,leu,ala,thr,ser,val,val
         ELS42853_A5,ELS42853_A6,ELS42853_A7,ELS42853_A8,ELS42850_A1,ELS42847_A1,ELS42847_A2,ELS428
47_A3    ala,val,leu,ala,thr,ser,N/A,N/A   ala,val,leu,ala,thr,ser,dab,dab
4.       BA000019 c9      Nostoc sp. PCC 7120 DNA, complete genome.  5.96713675841
         0.333333333333   Val,Val,Thr/Ser,Thr/Ser,Ala,Ile/Leu,Val,Gly
         gly,val,leu,ala,ser,ser,val
         BAB74343_A1,BAB74348_A1,BAB74346_A1,BAB74346_A2,BAB74347_A1,BAB74342_A1,BAB74342_A2
         gly,tyr,gly,ala,ser,ser,val       gly,tyr,gly,ala,ser,ser,val
5.       CP003235 c6      Paenibacillus mucilaginosus 3016, complete genome.  5.78639323983
         0.714285714286   Val,Val,Thr/Ser,Thr/Ser,Ala,Ile/Leu,Val,Gly
         gly,val,leu,ala,ser,ser,val,val
         AFC30714_A2,AFC30714_A3,AFC30709_A1,AFC30709_A2,AFC30715_A1,AFC30715_A2,AFC30715_A3,AFC307
15_A4    gly,val,leu,cys,ser,ser,val,cys   gly,val,leu,cys,ser,ser,val,cys
…
```

When using the command

```
python nrp2path.py --db genbank.ppd --taxonomy Streptomyces
Val,Val,Thr/Ser,Thr/Ser,Ala,Ile/Leu,Val,Gly
```

to delimit the search to Streptomyces BGCs, the difference between the stendomycin BGC and the runners-up becomes much more pronounced:

```
Sequence tag 1: Val,Val,Thr/Ser,Thr/Ser,Ala,Ile/Leu,Val,Gly
Rank     Accession        Cluster nr       Description      Nrp2Path Score   Colinearity Score
         Sequence tag     Matching AA      Matching domains NRPSPredictor2 code predictions
         NRPSPredictor2 SVM predictions
1.       GG657754 c10     Streptomyces hygroscopicus ATCC 53653 genomic scaffold supercont1.1,
whole genome shotgun sequence.    7.53553517418    1.0
         Val,Val,Thr/Ser,Thr/Ser,Ala,Ile/Leu,Val,Gly        gly,val,ile/leu,ala,thr,thr,val,val
         EFL21631_A1,EFL21631_A2,EFL21631_A3,EFL21631_A4,EFL21631_A5,EFL21631_A6,EFL21631_A7,EFL216
32_A1    gly,val,N/A,ala,thr,thr,val,val   gly,val,val,ala,thr,thr,val,val
2.       AM746336 c1      Streptomyces collinus kirromycin biosynthesis gene cluster, strain Tu
365.     5.49641118813    0.2      Val,Val,Thr/Ser,Thr/Ser,Ala,Ile/Leu,Val,Gly
         thr,ser,ala,ile,val,gly
         CAN89656_A1,CAN89656_A2,CAN89663_A1,CAN89638_A1,CAN89662_A1,CAN89633_A1
         thr,ser,N/A,gly,val,gly   thr,ser,ser,gly,val,gly
3.       CM000950 c17     Streptomyces pristinaespiralis ATCC 25486 chromosome, whole genome
shotgun sequence.4.85734270255    0.25     Val,Val,Thr/Ser,Thr/Ser,Ala,Ile/Leu,Val,Gly
         ile/leu,ala,ser,thr,val   EDY67138_A1,EFH32277_A1,EFH32236_A1,EDY67106_A1,EDY67106_A2
         N/A,N/A,ser,thr,val       met,ser,ser,thr,abu
4.       AGSW01000262     c1       Streptomyces sp. W007 contig00280, whole genome shotgun
sequence.        4.59308354132    0.0      Val,Val,Thr/Ser,Thr/Ser,Ala,Ile/Leu,Val,Gly
         gly,val,ile,ala,thr,thr
```

```
        EHM24052_A1,EHM24082_A1,EHM24059_A1,EHM24066_A1,EHM24083_A1,EHM24087_A1
        gly,N/A,ile,N/A,thr,N/A   gly,dpg,ile,pro,thr,tyr
5.      AOPZ01000014      c1      Streptomyces aurantiacus JA 4570 Seq14, whole genome shotgun
sequence.       4.48304176461     0.6     Val,Val,Thr/Ser,Thr/Ser,Ala,Ile/Leu,Val,Gly
        gly,val,ile,ala,ser,ser
        EPH46598_A1,EPH46610_A1,EPH46597_A1,EPH46597_A2,EPH46596_A1,EPH46596_A2
        gly,N/A,gly,ala,N/A,ser   val,val,gly,ala,ser,ser
```

The size of the tag can be varied, by taking a contiguous substring out of the original tag, e.g. T(S)-T(S)-A-I(L). Depending on the specific tag and tag size chosen, you will get different search results. Figure 2 of the manuscript shows the average result for all possible tags of sizes 2-8 when searching either at the genome level (within *Streptomyces hygroscopicus* ATCC 53653) or at the genus level (within *Streptomyces*) using the --taxonomy option available from the command-line.

Instead of the extended amino acid tag, one might also want to use the full original mass shift sequence, from which a larger number of tags are generated (including more rare amino acids). This also removes the need for a manual interpretation step. The mass shift sequence obtained for stendomycin by Kersten et al. is ’99-99-83-83-71-113-99-57’. Hence, you can run nrp2path with, e.g., the following command:

```
python nrp2path.py --db genbank.ppd --taxonomy Streptomyces
99,99,83,83,71,113,99,57
```

This will yield the following results:

```
Sequence tag 1: 99,99,83,83,71,113,99,57
Rank    Accession        Cluster nr       Description      Nrp2Path Score   Colinearity Score
        Sequence tag     Matching AA      Matching domains NRPSPredictor2 code predictions
        NRPSPredictor2 SVM predictions
1.      GG657754 c10     Streptomyces hygroscopicus ATCC 53653 genomic scaffold supercont1.1,
whole genome shotgun sequence.    12.1692710096    1.0      99,99,83,83,71,113,99,57
        gly,val,ile/leu/val,ala,thr,thr,val,val
        EFL21631_A1,EFL21631_A2,EFL21631_A3,EFL21631_A4,EFL21631_A5,EFL21631_A6,EFL21631_A7,EFL216
32_A1   gly,val,N/A,ala,thr,thr,val,val   gly,val,val,ala,thr,thr,val,val
2.      GG657754 c7      Streptomyces hygroscopicus ATCC 53653 genomic scaffold supercont1.1,
whole genome shotgun sequence.    9.43698943739    0.571428571429   99,99,83,83,71,113,99,57
        iva,iva,thr,thr/ser,ala,val,ala,gly
        EFL21149_A1,EFL21153_A1,EFL21152_A1,EFL21152_A2,EFL21150_A1,EFL21150_A2,EFL21150_A3,EFL211
50_A4   N/A,N/A,thr,N/A,ala,val,ala,N/A   tyr,tyr,thr,glu,ser,val,ala,tyr
3.      JX508597 c1      Streptomyces griseoviridis culture-collection NRRL:2427 griseoviridin and
viridogrisein Sgv gene cluster, complete sequence. 9.31003856982    0.333333333333
        99,99,83,83,71,113,99,57  ala,pro,gly,dhb,ser,ala,iva
        AGN74898_A1,AGN74886_A1,AGN74892_A1,AGN74880_A1,AGN74895_A1,AGN74885_A1,AGN74885_A2
        cys,N/A,gly,pip,ser,gly,N/A       cys,tyr,gly,sal,ser,gly,val
4.      CP006567 c39     Streptomyces rapamycinicus NRRL 5491 genome.       8.62839006724
        0.857142857143   99,99,83,83,71,113,99,57  gly,val,val,gly,thr,thr/ser,val,iva
        AGP60631_A2,AGP60631_A3,AGP60631_A4,AGP60631_A5,AGP60700_A1,AGP60700_A2,AGP60700_A3,AGP607
00_A4   ala,val,ala,N/A,thr,N/A,val,N/A   ala,val,ala,tyr,thr,glu,val,tyr
5.      NZ_ALNP01000029  c1      Streptomyces sp. AA0539 contig00029, whole genome shotgun
sequence.       8.58449212445    0.857142857143   99,99,83,83,71,113,99,57
        val,val,thr,thr,ala,leu/val,val,gly
        WP_020699878_A1,WP_020699878_A2,WP_020699878_A3,WP_020699878_A4,WP_020699878_A5,WP_0194359
44_A1,WP_019435944_A2,WP_019435944_A3     val,val,thr,thr,val,N/A,val,val
        val,val,thr,thr,val,gly,val,val
```

Note that the score is different from the score for the previously used tag ”Val,Val,Thr/Ser,Thr/Ser,Ala,Ile/Leu,Val,Gly”. The reason for this is that there is a larger number of redundant amino acids in the tag: the mass shift tag 99,99,83,83,71,113,99,57 in fact translates to “Val/Ala/Iva/Ile/Leu,Val/Ala/Iva/Ile/Leu,Thr/Dhb/Ser,Thr/Dhb/Ser,Ala/B-Ala/Gly,Abu/Pro/Ile/Leu/Val,Val/Ala/Iva/Ile/Leu,Gly”. The consequence is that in the new situations more tag positions are calculated with baseline P(A) values, as the amino acid is not considered an observation.

2. Eighteen recently discovered nonribosomal peptides.

A second data set that can be used to test Pep2Path is constituted by the benchmarking dataset of 18 recently discovered nonribosomal peptides outlined in Supplementary Table 1 of the manuscript.

Peptide	(Sub)phylum	Species	Gene cluster (NCBI accession + antiSMASH gene cluster number)	NRP search tag
Micropeptin	Cyanobacteria	*Microcystis Aeruginosa* K-139	AB481215_c1	Asp-Thr-Arg-Thr-Ile-Tyr-Ile
Skyllamycin	Actinobacteria	*Streptomyces* sp. Acta 2897	JF430460_c1	Ala-Asp-Gly-Phe-Pro-Tyr-Trp-Gly-Leu-Leu
Pyridomycin	Actinobacteria	*Streptomyces pyridomyceticus* NRRL B-2517	HM436809_c1	Pip-Thr-Ala-Xxx-Xxx
Althiomycin	Deltaproteobacteria	*Myxococcus xanthus* DK897	FR831800_c1	Gly-Cys-Ser-Cys-Gly
Hormaomycin	Actinobacteria	*Streptomyces griseoflavus* w-384	HQ542230_c1	Pro-Ala-Thr-Phe-Ala-Phe-Ile-Pro
Koranimine	Firmicutes	*Bacillus* sp. NK 2003	JF828091_c1	Thr-Leu-Leu-Phe-Val-Phe-Val
Nostophycin	Cyanobacteria	*Nostoc* sp. Strain 152	JF430079_c1	Gln-Gly-Pro-Phe-Ile-Pro
Arylomycin	Actinobacteria	*Streptomyces roseosporus* NRRL 11379	NZ_ABYX01000222_c1	Ser-Ala-Gly-Hpg-Ala-Tyr
Myxoprincomide	Deltaproteobacteria	*Myxococcus xanthus* DK 1622	NC_008095_c11	Ser-Leu-Val-Ser-Val-Ser-Tyr-Lys-Ala
Nodularin	Cyanobacteria	*Nostoc* sp. 73.1	JF342711_c1	Asp-Arg-Xxx-Glu-Ser
Napsamycin	Actinobacteria	*Streptomyces* sp. DSM5940	HQ287563_c1	Tyr-Met-Dab-Trp
Cupriachelin	Betaproteobacteria	*Ralstonia eutropha* H16	NC_008314_c1	Xxx-Asp-Dab-Asp-Gly-Orn
Caerulomycin	Actinobacteria	*Actinoalloteichus* sp. WH1-2216-6	JF419316_c1	Cys-Thr
Collismycin	Actinobacteria	*Streptomyces* sp. CS40	HE575208_c1	Cys-Leu
Rhodochelin	Actinobacteria	*Rhodococcus jostii* RHA1	NC_008268_c8	Thr-Orn
Nocobactin	Actinobacteria	*Nocardia farcinica* IFM 10152	NC_006361_c3	Lys-Lys-Thr
Paenibactin	Firmicutes	*Paenibacillus eigii* B69	HQ668144_c1	Dhb-Ala-Thr
Mycobactin	Actinobacteria	*Mycobacterium tuberculosis* H37Rv	NC_000962_c9	Sal-Thr-Lys

This data set contains both peptides with long sequence tags (for which it is easier to identify their BGCs) and peptides with short sequence tags (for which it is more difficult to identify their BGCs).
Let us look at one example of each.
The skyllamycin molecule consists of 10 amino acids, which together form a sequence tag 'Ala-Asp-Gly-Phe-Pro-Tyr-Trp-Gly-Leu-Leu'. To search for the BGC of this molecule in the entire GenBank database, we use the following command:

```
python nrp2path.py --db genbank.ppd Ala,Asp,Gly,Phe,Pro,Tyr,Trp,Gly,Leu,Leu
```

This immediately leads to the identification of a very good hit which stands out from the lower-ranking hits (score of 5.48 compared to other scores <4.32):

```
Sequence tag 1: Ala,Asp,Gly,Phe,Pro,Tyr,Trp,Gly,Leu,Leu
1.      JF430460 c1      Streptomyces sp. Acta 2897 skyllamycin biosynthetic gene cluster,
complete sequence.       5.4825289332     1.0      ala,asp,gly,phe,pro,tyr,trp,gly,leu,leu
        ala,asp,gly,phe,pro,tyr,trp,gly,leu,leu
        AEA30272_A2,AEA30272_A3,AEA30272_A4,AEA30273_A1,AEA30273_A2,AEA30273_A3,AEA30273_A4,AEA302
74_A1,AEA30274_A2,AEA30274_A3     ala,asn,gly,phe,pro,N/A,N/A,gly,N/A,N/A
        ala,asp,gly,phe,pro,phe,trp,gly,tyr,tyr
2.      ANKQ01000001     c4      Microcystis aeruginosa TAIHU98 contig1, whole genome shotgun
sequence.        4.31423570852    0.6      ala,asp,gly,phe,pro,tyr,trp,gly,leu,leu
        asp,gly,phe,pro,tyr,trp
        ELP56809_A1,ELP56284_A1,ELP56284_A2,ELP57196_A1,ELP57196_A2,ELP55719_A1
        N/A,val,tyr,pro,tyr,tyr   leu,val,tyr,pro,tyr,tyr
3.      NZ_AOUG01000020  c1      Burkholderia pseudomallei NCTC 13392 NCTC13392_28, whole genome
shotgun sequence.4.13566642421    0.0      ala,asp,gly,phe,pro,tyr,trp,gly,leu,leu    ala,asp,gly
        WP_017844181_A1,WP_017844174_A1,WP_017844180_A1    ala,asp,gly      ala,asp,gly
4.      CP006469 c7      Burkholderia pseudomallei MSHR305 chromosome 1, complete sequence.
        4.13566642421    0.0      ala,asp,gly,phe,pro,tyr,trp,gly,leu,leu    ala,asp,gly
        AGR68580_A1,AGR67582_A1,AGR69939_A1        ala,asp,gly      ala,asp,gly
5.      CP003782 c10     Burkholderia pseudomallei BPC006 chromosome II, complete sequence.
        4.13566642421    0.0      ala,asp,gly,phe,pro,tyr,trp,gly,leu,leu    ala,asp,gly
        AFR19519_A1,AFR19491_A1,AFR19517_A1        ala,asp,gly      ala,asp,gly
```

…

On the contrary, when we use the very short and much more unspecific 'Cys-Thr' sequence tag of caerulomycin as a query for a GenBank-wide search, using the command

```
python nrp2path.py --db genbank.ppd Cys,Thr
```

we get a wide range of almost equally possible hits. Here are the top five hits for this search, with scores between 4.40 and 4.53:

```
Sequence tag 1: Cys,Thr
1.      CP001630 c11      Actinosynnema mirum DSM 43827, complete genome.     4.52672830483    0.0
        cys,thr  cys,thr  ACU37567_A2,ACU37564_A1   cys,thr  cys,thr
2.      CAGD01000003     c1      Brevibacillus laterosporus GI-9 WGS project CAGD00000000 data,
contig BLGIcontig03, whole genome shotgun sequence. 4.48198383548    0.0      thr,cys  thr,cys
        CCF13030_A1,CCF12997_A1   thr,cys  thr,cys
3.      AFRV01000004     c1      Brevibacillus laterosporus LMG 15441 BRLAcontig4c, whole genome
shotgun sequence. 4.48198383548    0.0      thr,cys  thr,cys  EGP33003_A1,EGP32975_A1   thr,cys
        thr,cys
4.      KF264553 c1      Uncultured bacterium esnapd14 genomic sequence.     4.45274478818    0.0
        cys,thr  cys,thr  AGS49688_A2,AGS49687_A1   cys,thr  cys,thr
5.      AJGF01000005     c2      Mycobacterium abscessus M93 Contig05, whole genome shotgun
sequence.        4.39663391462    0.0      cys,thr  cys,thr  EIC67550_A2,EIC67544_A1   cys,thr
        cys,thr
```

If we have taxonomic information on the organism from which the peptide was derived, this helps in identifying the correct gene cluster nonetheless. In this case, we know that the molecule derives from the genus *Actinoalloteichus*, so we can use this to do a more specific search:

```
python nrp2path.py --db genbank.ppd --taxonomy Actinoalloteichus Cys,Thr
```

Now, the top five contains three copies of the actual caerulomycin gene cluster in different *Actinoalloteichus* species, with equal scores (4.15). The next hits, which correspond to altogether unrelated gene clusters, have scores of just 2.39 and 0.12:

```
Sequence tag 1: Cys,Thr
1.      NZ_AGVX02000004  c1      Actinoalloteichus spitiensis RMV-1378 Contig4, whole genome
shotgun sequence. 4.15293228248    1.0      cys,thr  cys,thr  WP_016696705_A1,WP_016696706_A1
        cys,thr  cys,thr
2.      JQ687072 c1      Actinoalloteichus cyanogriseus strain NRRL B-2194 methyltransferase
(caeG2), transporter (caeH3), transcriptional regulator (caeI2), ABC transporter (caeH1), ABC
transporter (caeH2), acyl-CoA dehydrogenase (caeB5), methyltransferase (caeG1), aminotransferase
(caeC), FAD-dependentt oxidoreductase (caeB6), NrpS (caeA1), L-lysine 2-amino transferase (caeP1),
FAD-dependent oxidoreductase (caeP2), PKS/NrpS (caeA2), NrpS (caeA3), acyl-CoA dehydrogenase
(caeB1), thioesterase (caeA4), LuxR family two component transcriptional regulator (caeI1),
amidohydrolase (caeD), AMP-dependent ligase (caeF), aldehyde dehydrogenase (caeB2), FAD-dependent
oxidoreductase (caeB3), F420-dependent NADP oxidoreductase (caeB4), transcriptional regulator
(caeI3), and monooxygenase (caeB7) genes, complete cds.     4.15293228248    1.0      cys,thr
        cys,thr  AFK24516_A1,AFK24517_A1   cys,thr  cys,thr
3.      JF419316 c1      Actinoalloteichus sp. WH1-2216-6 putative formate acetyltransferase gene,
partial cds; 50S ribosomal protein and ubiquinone/menaquinone biosynthesis melthyltransgerase
genes, complete cds; caerulomycin A biosynthetic gene cluster, complete sequence; and F420-
dependent NADP oxidoreductase coenzyme gene, partial cds.   4.15293228248    1.0      cys,thr
        cys,thr  AFD30954_A1,AFD30953_A1   cys,thr  cys,thr
4.      NZ_AGVX02000207  c1      Actinoalloteichus spitiensis RMV-1378 Contig207, whole genome
shotgun sequence. 2.39341063725    1.0      cys,thr  cys,thr  WP_016698708_A1,WP_016698709_A1
        N/A,thr  trp,thr
5.      NZ_AGVX02000192  c1      Actinoalloteichus spitiensis RMV-1378 Contig192, whole genome
shotgun sequence. 0.119351846221   N/A      cys,thr  cys      WP_016698577_A1  leu      val
```

Table 1 outlines the results of a procedure in which all possible (sub)tags of sizes 2-8 from the set of 18 NRPs were used on 50 randomly generated search spaces of sizes 5 to 100.

3. Nine recently discovered RiPPs.

Finally, the set of search tags for nine *Streptomyces* RiPPs outlined in Table 3 of the manuscript offer a way to test the ripp2path script.

Peptide	Search tag	Genome
SSV-2083	I(L)GA(C)GTA(C)WI(L)A(C)V	*Streptomyces sviceus* ATCC 20983
SGR-1832	AVAQ(K)FVI(L)Q(K)GSTI(L)	*Streptomyces griseus* IFO 13350
SCO-2138	VHFVGWI(L)	*Streptomyces coelicolor* A3(2)
SLI-2138	GI(L)VHFVGWI(L)	*Streptomyces lividans* TK24
SWA-2138	I(L)AGI(L)VHFI(L)GWI(L)	*Streptomyces* sp. E14 (WASP)
SRO15-2005	YWSRRI(L)I(L)	*Streptomyces roseosporus* NRRL 15998
SRO15-2212	VVI(L)S(C)T	*Streptomyces roseosporus* NRRL 15998
SRO15-3108	AS(C)ATVTI(L)	*Streptomyces roseosporus* NRRL 15998
SAL-2242	VTI(L)S(C)T	*Streptomyces albus* J1074

The genomes of the corresponding *Streptomyces* strains have been included within the 'RiPP_genomes' subdirectory. One can run ripp2path on these peptide search tags, by e.g. using the command

```
python ripp2path.py --tags Val,His,Phe,Val,Gly,Trp,Ile/Leu --seq
TestData/RiPP_genomes/S_coelicolor.gbk
```

for the SCO-2138 peptide and its VHFVGWI(L) search tag.
This leads to a unique match in the *Streptomyces coelicolor* A3(2) genome, as can be seen in the following results:

```
Sequence tags: Val,His,Phe,Val,Gly,Trp,Ile/Leu
Rank   Accession     Start position(s)   Strand Query tag sequence  Matching
sequence      % Identity
1      AL645882      2625565      +      VHFVGWL      VHFVGWL      100
2      AL645882      2625565      +      VHFVGWL      VHFVGWI      85
3      AL645882      4858516      -      VGFVGWI      VHFVGWI      85
4      AL645882      2233450      -      WHFVGQL      VHFVGWL      71
5      AL645882      5710464      -      VWFIGWL      VHFVGWL      71
6      AL645882      1290558      -      VVGVGWL      VHFVGWL      71
7      AL645882      6742392      -      VVFLGWL      VHFVGWL      71
8      AL645882      2505024      -      VVEVGWL      VHFVGWL      71
9      AL645882      7271690      +      VTVVGWL      VHFVGWL      71
10     AL645882      7455987      -      VTFVGAL      VHFVGWL      71
...
```

The right column in Table 3 of the manuscript shows the number of 100% matches obtained with each of the nine RiPP extended sequence tags in the table at the top of this page.
