# Supplementary material for: Pep2Path: Automated Mass Spectrometry-Guided Genome Mining of Peptidic Natural Products
Source: PLoS Comput Biol. 2014 Sep 4;10(9):e1003822. doi: 10.1371/journal.pcbi.1003822 (PMC4154637; doi:10.1371/journal.pcbi.1003822)
Supplement: Figure S1 — Gene cluster and predicted possible biosynthetic scheme for the production of tripropeptin-like molecules by Collimonas fungivorans Ter331. The gene cluster was identified by Pep2Path based on the raw amino acid sequence of compounds originally purified from Lysobacter sp. BMK333-48F3. The outline of the gene cluster and its encoded NRPS assembly-line above shows that the architecture of the gene cluster is consistent with the chemical structure of molecules highly similar to the tripropeptins that have been identified in Lysobacter. This BGC has eight NRPS modules, of which seven gave NRPSPredictor2 predictions exactly matching the tripropeptin A sequence in the right order, while the eighth prediction was only a near miss (ornithine predicted instead of arginine). This indicates that such a gene cluster might have undergone horizontal gene transfer at least once, from one subphylum to another. The peptides actually produced by Collimonas might have small differences in chemistry, compared to the Lysobacter tripropeptins, due to slight variations and/or promiscuity in the tailoring reactions and substrate acceptance. (PDF) [file pcbi.1003822.s002.pdf]

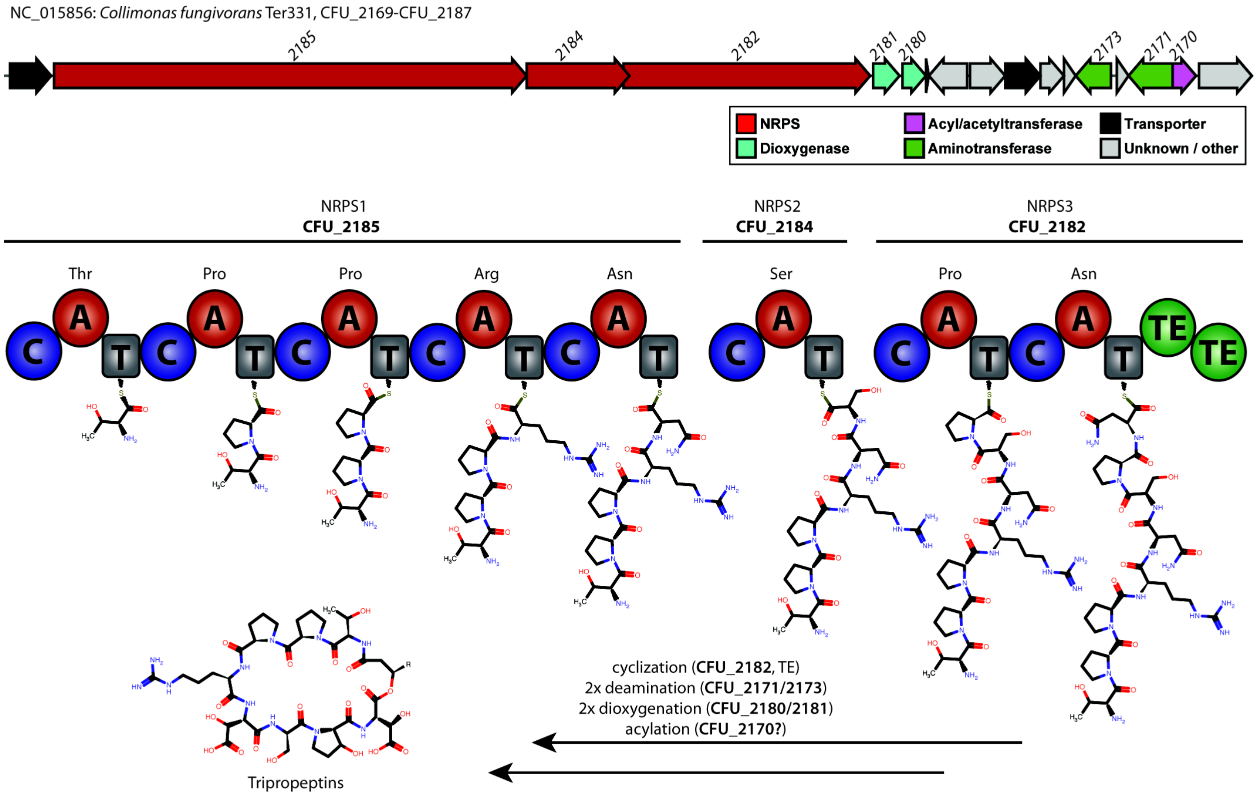

Figure S1: Gene cluster and predicted possible biosynthetic scheme for the production of tripropeptin-like molecules by *Collimonas fungivorans* Ter331. The gene cluster was identified by Pep2Path based on the raw amino acid sequence of compounds originally purified from *Lysobacter* sp. BMK333-48F3. The outline of the gene cluster and its encoded NRPS assembly-line above shows that the architecture of the gene cluster is consistent with the chemical structure of molecules highly similar to the tripropeptins that have been identified in *Lysobacter*. This BGC has eight NRPS modules, of which seven gave NRPSPredictor2 predictions exactly matching the tripropeptin A sequence in the right order, while the eighth prediction was only a near miss (ornithine predicted instead of arginine). This indicates that such a gene cluster might have undergone horizontal gene transfer at least once, from one subphylum to another. The peptides actually produced by *Collimonas* might have small differences in chemistry, compared to the *Lysobacter* tripropeptins, due to slight variations and/or promiscuity in the tailoring reactions and substrate acceptance.
