# Supplementary material for: Pep2Path: Automated Mass Spectrometry-Guided Genome Mining of Peptidic Natural Products
Source: PLoS Comput Biol. 2014 Sep 4;10(9):e1003822. doi: 10.1371/journal.pcbi.1003822 (PMC4154637; doi:10.1371/journal.pcbi.1003822)
Supplement: Table S1 — NRP2Path benchmarking dataset. The table displays 18 recently experimentally characterized NRPs and their biosynthetic gene clusters used for benchmarking NRP2Path. Rare amino acids that are not covered by the Pep2Path translation table are marked as ‘Xxx’. (PDF) [file pcbi.1003822.s003.pdf]

Peptide	(Sub)phylum	Species	Gene cluster (NCBI accession + antiSMASH gene cluster number)	NRP search tag
Micropeptin	Cyanobacteria	*Microcystis Aeruginosa* K-139	AB481215_c1	Asp-Thr-Arg-Thr-Ile-Tyr-Ile
Skyllamycin	Actinobacteria	*Streptomyces* sp. Acta 2897	JF430460_c1	Ala-Asp-Gly-Phe-Pro-Tyr-Trp-Gly-Leu-Leu
Pyridomycin	Actinobacteria	*Streptomyces pyridomyceticus* NRRL B-2517	HM436809_c1	Pip-Thr-Ala-Xxx-Xxx
Althiomycin	Deltaproteobacteria	*Myxococcus xanthus* DK897	FR831800_c1	Gly-Cys-Ser-Cys-Gly
Hormaomycin	Actinobacteria	*Streptomyces griseoflavus* w-384	HQ542230_c1	Pro-Ala-Thr-Phe-Ala-Phe-Ile-Pro
Koranimine	Firmicutes	*Bacillus* sp. NK 2003	JF828091_c1	Thr-Leu-Leu-Phe-Val-Phe-Val
Nostophycin	Cyanobacteria	*Nostoc* sp. Strain 152	JF430079_c1	Gln-Gly-Pro-Phe-Ile-Pro
Arylomycin	Actinobacteria	*Streptomyces roseosporus* NRRL 11379	NZ_ABYX01000222_c1	Ser-Ala-Gly-Hpg-Ala-Tyr
Myxoprincomide	Deltaproteobacteria	*Myxococcus xanthus* DK 1622	NC_008095_c11	Ser-Leu-Val-Ser-Val-Ser-Tyr-Lys-Ala
Nodularin	Cyanobacteria	*Nostoc* sp. 73.1	JF342711_c1	Asp-Arg-Xxx-Glu-Ser
Napsamycin	Actinobacteria	*Streptomyces* sp. DSM5940	HQ287563_c1	Tyr-Met-Dab-Trp
Cupriachelin	Betaproteobacteria	*Ralstonia eutropha* H16	NC_008314_c1	Xxx-Asp-Dab-Asp-Gly-Orn
Caerulomycin	Actinobacteria	*Actinoalloteichus* sp. WH1-2216-6	JF419316_c1	Cys-Thr
Collismycin	Actinobacteria	*Streptomyces* sp. CS40	HE575208_c1	Cys-Leu
Rhodochelin	Actinobacteria	*Rhodococcus jostii* RHA1	NC_008268_c8	Thr-Orn
Nocobactin	Actinobacteria	*Nocardia farcinica* IFM 10152	NC_006361_c3	Lys-Lys-Thr
Paenibactin	Firmicutes	*Paenibacillus eigii* B69	HQ668144_c1	Dhb-Ala-Thr
Mycobactin	Actinobacteria	*Mycobacterium tuberculosis* H37Rv	NC_000962_c9	Sal-Thr-Lys

Table S1: NRP2Path benchmarking dataset. The table displays 18 recently experimentally characterized NRPs and their biosynthetic gene clusters used for benchmarking NRP2Path. Rare amino acids that are not covered by the Pep2Path translation table are marked as ‘Xxx’.
